# Satisfaction with nursing care: influence of sociodemographic factors on a sample of hospitalised children

**DOI:** 10.1080/07853890.2021.1896876

**Published:** 2021-09-28

**Authors:** Fernanda Loureiro, Zaida Charepe

**Affiliations:** a Escola Superior de Saúde Egas Moniz, Caparica, Portugal; b Instituto Ciências da Saúde, CIIS-Centro de Investigação Interdisciplinar em Saúde, UCP/ICS, Universidade Católica Portuguesa, Lisboa, Portugal

## Abstract

**Introduction:**

Patient satisfaction is identified as an indicator of the right to health [1]. Traditionally, children’s satisfaction with health care is not regularly accessed [2] however, in recent years, it has been increasingly studied [3]. This study aims to identify if sociodemographic factors, such as sex, age and reason for hospitalisation, influences satisfaction with nursing care, in a sample of school-aged children (7–11 years).

**Materials and methods:**

An observational, cross-sectional, exploratory-descriptive study with a non-probabilistic and accidental sample was performed. Data were collected through the “Children Care Quality at Hospital” instrument, after translation and validation to Portuguese. The instrument includes three domains: nurse characteristics, nursing activities and nursing environment. Also, children were asked to rate global satisfaction with nursing care from 1 (less satisfied) to 5 (more satisfied). Statistical analysis was performed using SPSS statistical tool (version 24.0). Authorisation was obtained from National Data Protection Commission as well as ethics committees in each of the 6 health institutions were the study was applied.

**Results:**

The sample (*n* = 252) includes mainly boys (52.8%, *n* = 133) with 8.9 years (SD = 1.4) as mean age and most children had unscheduled admissions (84.6%; *n* = 209). Global nursing care (1–5) was rated with a score of 4.51 (SD = 0.645). There was no significant difference between sex (*t*=–0.86; *p* > .05), age (*r*_s_=–0.49; *p* > .05) or scheduled/unscheduled admissions (*t*=–0.59; *p* > .05) and the score attributed by children.

**Discussion and conclusions:**

In this sample, school-aged children are satisfied with nursing care provided during hospitalisation. Sociodemographic factors seem to have effect on overall satisfaction in previous studies with better scores of satisfaction in: older patients [4], male patients [4,5] and unscheduled admissions [6]. Nevertheless, this was not verified in our sample. We suggest that further studies should be developed with larger samples and different group age.
